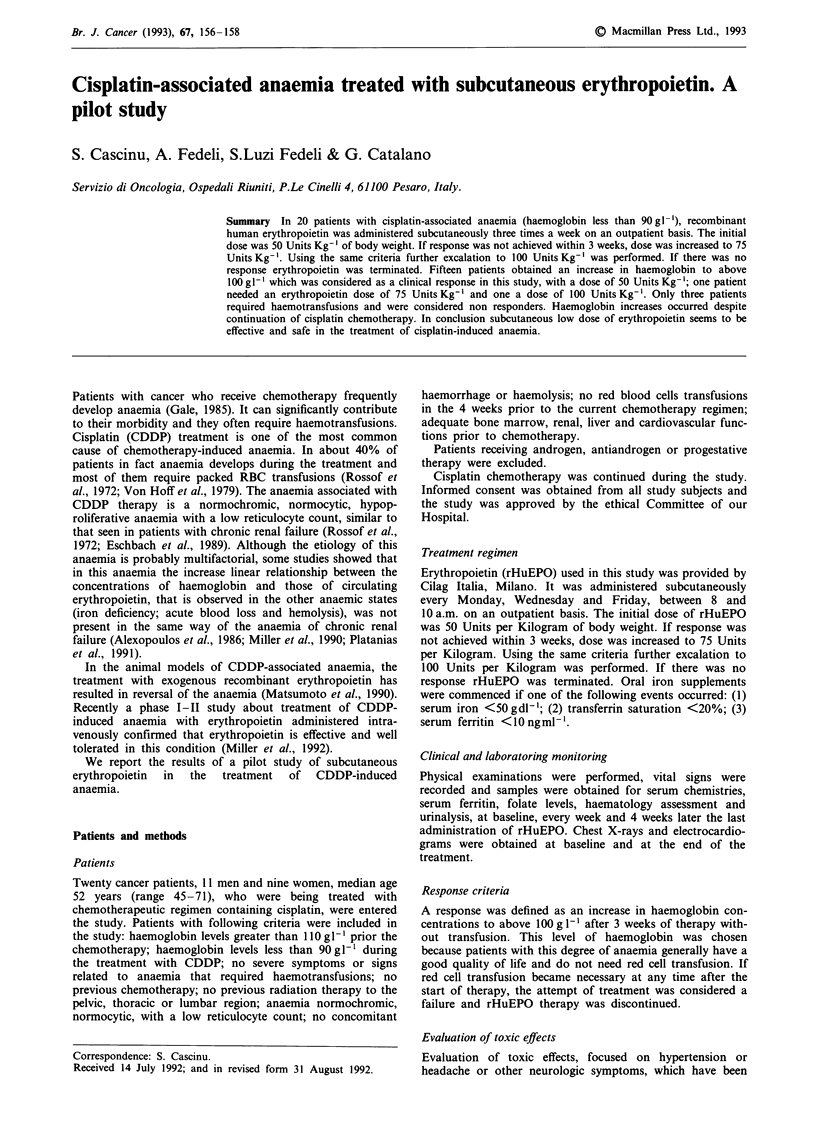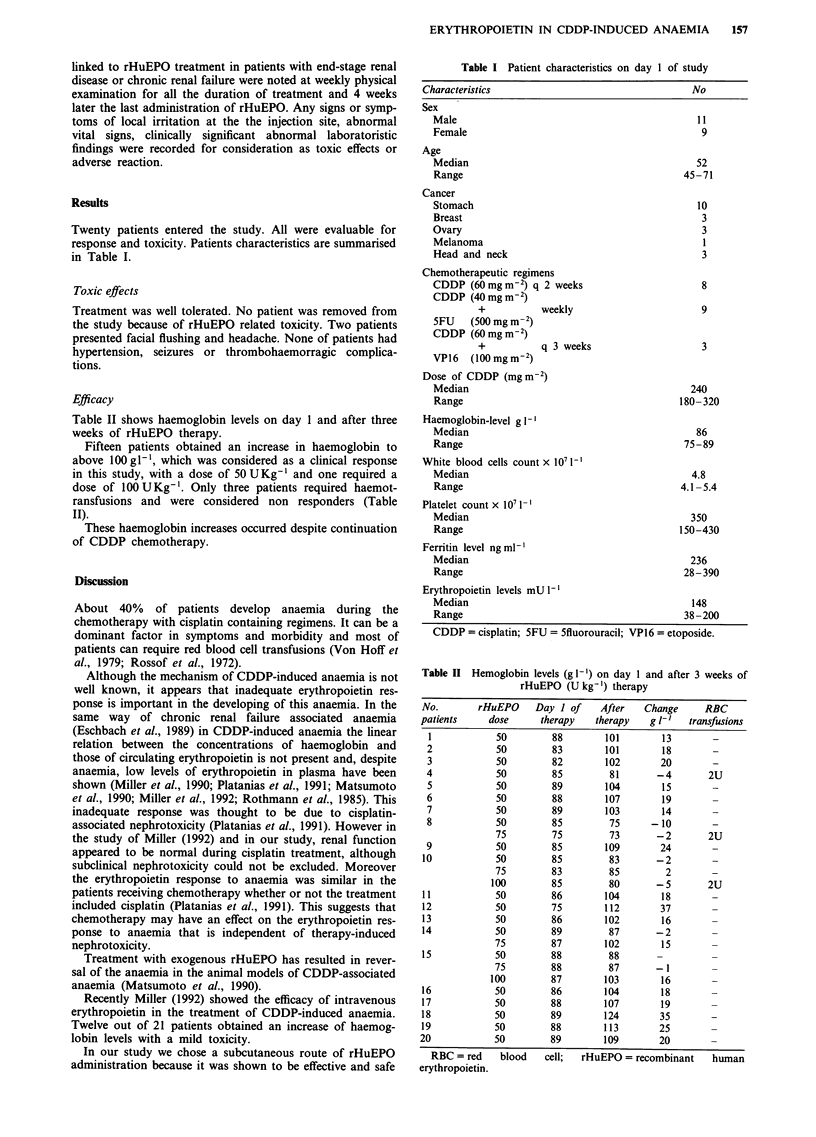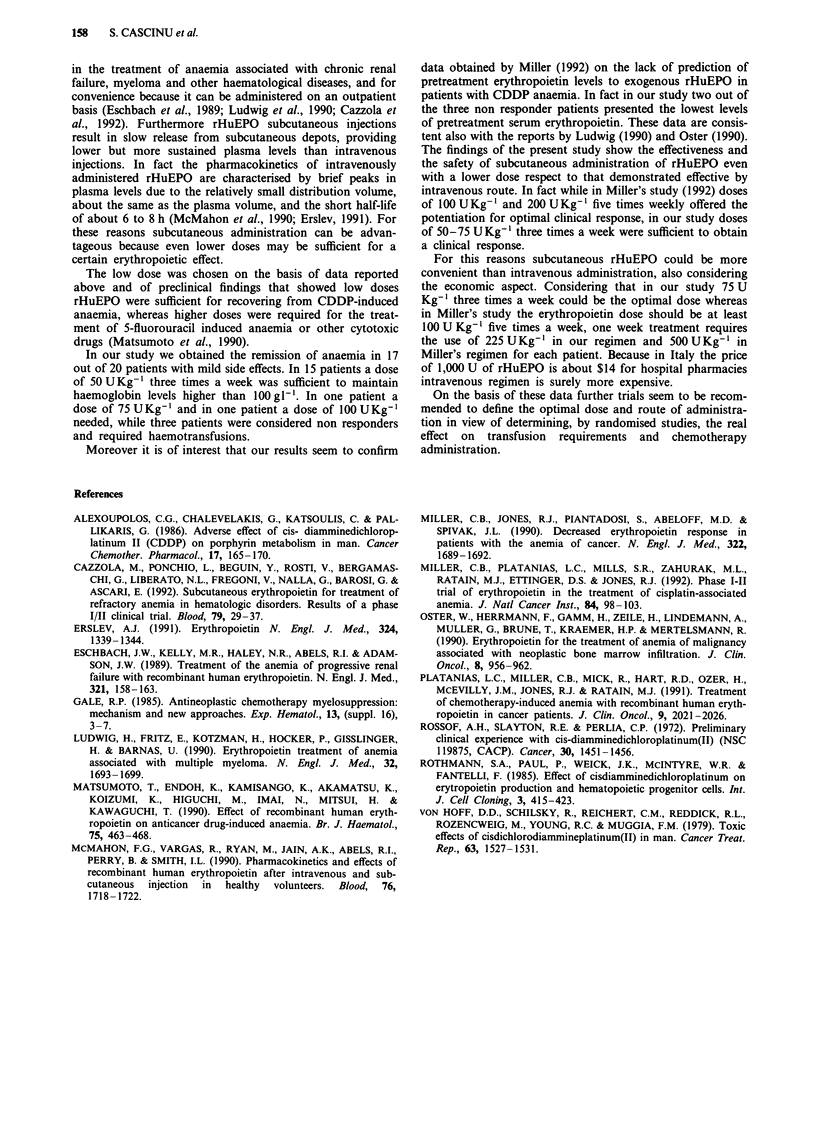# Cisplatin-associated anaemia treated with subcutaneous erythropoietin. A pilot study.

**DOI:** 10.1038/bjc.1993.27

**Published:** 1993-01

**Authors:** S. Cascinu, A. Fedeli, S. L. Fedeli, G. Catalano

**Affiliations:** Servizio di Oncologia, Ospedali Riuniti, Pesaro, Italy.

## Abstract

In 20 patients with cisplatin-associated anaemia (haemoglobin less than 90 gl-1), recombinant human erythropoietin was administered subcutaneously three times a week on an outpatient basis. The initial dose was 50 Units Kg-1 of body weight. If response was not achieved within 3 weeks, dose was increased to 75 Units Kg-1. Using the same criteria further escalation to 100 Units Kg-1 was performed. If there was no response erythropoietin was terminated. Fifteen patients obtained an increase in haemoglobin to above 100 gl-1 which was considered as a clinical response in this study, with a dose of 50 Units Kg-1; one patient needed an erythropoietin dose of 75 Units Kg-1 and one a dose of 100 Units Kg-1. Only three patients required haemotransfusions and were considered non responders. Haemoglobin increases occurred despite continuation of cisplatin chemotherapy. In conclusion subcutaneous low dose of erythropoietin seems to be effective and safe in the treatment of cisplatin-induced anaemia.


Br. J. Cancer (1993), 67, 156–158 © Macmillan Press Ltd., 1993

### Cisplatin-associated anaemia treated with subcutaneous erythropoietin. A pilot study

S. Cascinu, A. Fedeli, S.Luzi Fedeli & G. Catalano

*Servizio di Oncologia, Ospedali Riuniti, P.Le Cinelli 4, 61100 Pesaro, Italy.*

**Summary** In 20 patients with cisplatin-associated anaemia (haemoglobin less than 90 $g l^{-1}$), recombinant human erythropoietin was administered subcutaneously three times a week on an outpatient basis. The initial dose was 50 Units $Kg^{-1}$ of body weight. If response was not achieved within 3 weeks, dose was increased to 75 Units $Kg^{-1}$. Using the same criteria further excalation to 100 Units $Kg^{-1}$ was performed. If there was no response erythropoietin was terminated. Fifteen patients obtained an increase in haemoglobin to above 100 $g l^{-1}$ which was considered as a clinical response in this study, with a dose of 50 Units $Kg^{-1}$; one patient needed an erythropoietin dose of 75 Units $Kg^{-1}$ and one a dose of 100 Units $Kg^{-1}$. Only three patients required haemotransfusions and were considered non responders. Haemoglobin increases occurred despite continuation of cisplatin chemotherapy. In conclusion subcutaneous low dose of erythropoietin seems to be effective and safe in the treatment of cisplatin-induced anaemia.

Patients with cancer who receive chemotherapy frequently develop anaemia (Gale, 1985). It can significantly contribute to their morbidity and they often require haemotransfusions. Cisplatin (CDDP) treatment is one of the most common cause of chemotherapy-induced anaemia. In about 40% of patients in fact anaemia develops during the treatment and most of them require packed RBC transfusions (Rossof *et al*., 1972; Von Hoff *et al*., 1979). The anaemia associated with CDDP therapy is a normochromic, normocytic, hypoproliferative anaemia with a low reticulocyte count, similar to that seen in patients with chronic renal failure (Rossof *et al*., 1972; Eschbach *et al*., 1989). Although the etiology of this anaemia is probably multifactorial, some studies showed that in this anaemia the increase linear relationship between the concentrations of haemoglobin and those of circulating erythropoietin, that is observed in the other anaemic states (iron deficiency; acute blood loss and hemolysis), was not present in the same way of the anaemia of chronic renal failure (Alexopoulos *et al*., 1986; Miller *et al*., 1990; Platanias *et al*., 1991).

In the animal models of CDDP-associated anaemia, the treatment with exogenous recombinant erythropoietin has resulted in reversal of the anaemia (Matsumoto *et al*., 1990). Recently a phase I–II study about treatment of CDDP-induced anaemia with erythropoietin administered intravenously confirmed that erythropoietin is effective and well tolerated in this condition (Miller *et al*., 1992).

We report the results of a pilot study of subcutaneous erythropoietin in the treatment of CDDP-induced anaemia.

### Patients and methods

### *Patients*

Twenty cancer patients, 11 men and nine women, median age 52 years (range 45–71), who were being treated with chemotherapeutic regimen containing cisplatin, were entered the study. Patients with following criteria were included in the study: haemoglobin levels greater than 110 $g l^{-1}$ prior the chemotherapy; haemoglobin levels less than 90 $g l^{-1}$ during the treatment with CDDP; no severe symptoms or signs related to anaemia that required haemotransfusions; no previous chemotherapy; no previous radiation therapy to the pelvic, thoracic or lumbar region; anaemia normochromic, normocytic, with a low reticulocyte count; no concomitant haemorrhage or haemolysis; no red blood cells transfusions in the 4 weeks prior to the current chemotherapy regimen; adequate bone marrow, renal, liver and cardiovascular functions prior to chemotherapy.

Patients receiving androgen, antiandrogen or progestative therapy were excluded.

Cisplatin chemotherapy was continued during the study. Informed consent was obtained from all study subjects and the study was approved by the ethical Committee of our Hospital.

### *Treatment regimen*

Erythropoietin (rHuEPO) used in this study was provided by Cilag Italia, Milano. It was administered subcutaneously every Monday, Wednesday and Friday, between 8 and 10 a.m. on an outpatient basis. The initial dose of rHuEPO was 50 Units per Kilogram of body weight. If response was not achieved within 3 weeks, dose was increased to 75 Units per Kilogram. Using the same criteria further excalation to 100 Units per Kilogram was performed. If there was no response rHuEPO was terminated. Oral iron supplements were commenced if one of the following events occurred: (1) serum iron $<50$ $g dl^{-1}$; (2) transferrin saturation $<20\%$; (3) serum ferritin $<10$ $ng ml^{-1}$.

### *Clinical and laboratoring monitoring*

Physical examinations were performed, vital signs were recorded and samples were obtained for serum chemistries, serum ferritin, folate levels, haematology assessment and urinalysis, at baseline, every week and 4 weeks later the last administration of rHuEPO. Chest X-rays and electrocardiograms were obtained at baseline and at the end of the treatment.

### *Response criteria*

A response was defined as an increase in haemoglobin concentrations to above 100 $g l^{-1}$ after 3 weeks of therapy without transfusion. This level of haemoglobin was chosen because patients with this degree of anaemia generally have a good quality of life and do not need red cell transfusion. If red cell transfusion became necessary at any time after the start of therapy, the attempt of treatment was considered a failure and rHuEPO therapy was discontinued.

### *Evaluation of toxic effects*

Evaluation of toxic effects, focused on hypertension or headache or other neurologic symptoms, which have been

Correspondence: S. Cascinu.
Received 14 July 1992; and in revised form 31 August 1992.

linked to rHuEPO treatment in patients with end-stage renal disease or chronic renal failure were noted at weekly physical examination for all the duration of treatment and 4 weeks later the last administration of rHuEPO. Any signs or symptoms of local irritation at the the injection site, abnormal vital signs, clinically significant abnormal laboratoristic findings were recorded for consideration as toxic effects or adverse reaction.

### Results

Twenty patients entered the study. All were evaluable for response and toxicity. Patients characteristics are summarised in Table I.

### *Toxic effects*

Treatment was well tolerated. No patient was removed from the study because of rHuEPO related toxicity. Two patients presented facial flushing and headache. None of patients had hypertension, seizures or thrombohaemorragic complications.

### *Efficacy*

Table II shows haemoglobin levels on day 1 and after three weeks of rHuEPO therapy.

Fifteen patients obtained an increase in haemoglobin to above 100 g $l^{-1}$, which was considered as a clinical response in this study, with a dose of 50 U $Kg^{-1}$ and one required a dose of 100 U $Kg^{-1}$. Only three patients required haemotransfusions and were considered non responders (Table II).

These haemoglobin increases occurred despite continuation of CDDP chemotherapy.

### Discussion

About 40% of patients develop anaemia during the chemotherapy with cisplatin containing regimens. It can be a dominant factor in symptoms and morbidity and most of patients can require red blood cell transfusions (Von Hoff *et al.*, 1979; Rossof *et al.*, 1972).

Although the mechanism of CDDP-induced anaemia is not well known, it appears that inadequate erythropoietin response is important in the developing of this anaemia. In the same way of chronic renal failure associated anaemia (Eschbach *et al.*, 1989) in CDDP-induced anaemia the linear relation between the concentrations of haemoglobin and those of circulating erythropoietin is not present and, despite anaemia, low levels of erythropoietin in plasma have been shown (Miller *et al.*, 1990; Platanias *et al.*, 1991; Matsumoto *et al.*, 1990; Miller *et al.*, 1992; Rothmann *et al.*, 1985). This inadequate response was thought to be due to cisplatin-associated nephrotoxicity (Platanias *et al.*, 1991). However in the study of Miller (1992) and in our study, renal function appeared to be normal during cisplatin treatment, although subclinical nephrotoxicity could not be excluded. Moreover the erythropoietin response to anaemia was similar in the patients receiving chemotherapy whether or not the treatment included cisplatin (Platanias *et al.*, 1991). This suggests that chemotherapy may have an effect on the erythropoietin response to anaemia that is independent of therapy-induced nephrotoxicity.

Treatment with exogenous rHuEPO has resulted in reversal of the anaemia in the animal models of CDDP-associated anaemia (Matsumoto *et al.*, 1990).

Recently Miller (1992) showed the efficacy of intravenous erythropoietin in the treatment of CDDP-induced anaemia. Twelve out of 21 patients obtained an increase of haemoglobin levels with a mild toxicity.

In our study we chose a subcutaneous route of rHuEPO administration because it was shown to be effective and safe

**Table I** Patient characteristics on day 1 of study

| *Characteristics* | | *No* |
|---|---|---|
| Sex | | |
| Male | | 11 |
| Female | | 9 |
| Age | | |
| Median | | 52 |
| Range | | 45–71 |
| Cancer | | |
| Stomach | | 10 |
| Breast | | 3 |
| Ovary | | 3 |
| Melanoma | | 1 |
| Head and neck | | 3 |
| Chemotherapeutic regimens | | |
| CDDP (60 mg $m^{-2}$) | q 2 weeks | 8 |
| CDDP (40 mg $m^{-2}$) + 5FU (500 mg $m^{-2}$) | weekly | 9 |
| CDDP (60 mg $m^{-2}$) + VP16 (100 mg $m^{-2}$) | q 3 weeks | 3 |
| Dose of CDDP (mg $m^{-2}$) | | |
| Median | | 240 |
| Range | | 180–320 |
| Haemoglobin-level g $l^{-1}$ | | |
| Median | | 86 |
| Range | | 75–89 |
| White blood cells count × $10^7$ $l^{-1}$ | | |
| Median | | 4.8 |
| Range | | 4.1–5.4 |
| Platelet count × $10^7$ $l^{-1}$ | | |
| Median | | 350 |
| Range | | 150–430 |
| Ferritin level ng $ml^{-1}$ | | |
| Median | | 236 |
| Range | | 28–390 |
| Erythropoietin levels mU $l^{-1}$ | | |
| Median | | 148 |
| Range | | 38–200 |

CDDP = cisplatin; 5FU = 5fluorouracil; VP16 = etoposide.

**Table II** Hemoglobin levels (g $l^{-1}$) on day 1 and after 3 weeks of rHuEPO (U $kg^{-1}$) therapy

| *No. patients* | *rHuEPO dose* | *Day 1 of therapy* | *After therapy* | *Change g $l^{-1}$* | *RBC transfusions* |
|---|---|---|---|---|---|
| 1 | 50 | 88 | 101 | 13 | – |
| 2 | 50 | 83 | 101 | 18 | – |
| 3 | 50 | 82 | 102 | 20 | – |
| 4 | 50 | 85 | 81 | –4 | 2U |
| 5 | 50 | 89 | 104 | 15 | – |
| 6 | 50 | 88 | 107 | 19 | – |
| 7 | 50 | 89 | 103 | 14 | – |
| 8 | 50 | 85 | 75 | –10 | – |
| | 75 | 75 | 73 | –2 | 2U |
| 9 | 50 | 85 | 109 | 24 | – |
| 10 | 50 | 85 | 83 | –2 | – |
| | 75 | 83 | 85 | 2 | – |
| | 100 | 85 | 80 | –5 | 2U |
| 11 | 50 | 86 | 104 | 18 | – |
| 12 | 50 | 75 | 112 | 37 | – |
| 13 | 50 | 86 | 102 | 16 | – |
| 14 | 50 | 89 | 87 | –2 | – |
| | 75 | 87 | 102 | 15 | – |
| 15 | 50 | 88 | 88 | – | – |
| | 75 | 88 | 87 | –1 | – |
| | 100 | 87 | 103 | 16 | – |
| 16 | 50 | 86 | 104 | 18 | – |
| 17 | 50 | 88 | 107 | 19 | – |
| 18 | 50 | 89 | 124 | 35 | – |
| 19 | 50 | 88 | 113 | 25 | – |
| 20 | 50 | 89 | 109 | 20 | – |

RBC = red blood cell; rHuEPO = recombinant human erythropoietin.

in the treatment of anaemia associated with chronic renal failure, myeloma and other haematological diseases, and for convenience because it can be administered on an outpatient basis (Eschbach et al., 1989; Ludwig et al., 1990; Cazzola et al., 1992). Furthermore rHuEPO subcutaneous injections result in slow release from subcutaneous depots, providing lower but more sustained plasma levels than intravenous injections. In fact the pharmacokinetics of intravenously administered rHuEPO are characterised by brief peaks in plasma levels due to the relatively small distribution volume, about the same as the plasma volume, and the short half-life of about 6 to 8 h (McMahon et al., 1990; Erslev, 1991). For these reasons subcutaneous administration can be advantageous because even lower doses may be sufficient for a certain erythropoietic effect.

The low dose was chosen on the basis of data reported above and of preclinical findings that showed low doses rHuEPO were sufficient for recovering from CDDP-induced anaemia, whereas higher doses were required for the treatment of 5-fluorouracil induced anaemia or other cytotoxic drugs (Matsumoto et al., 1990).

In our study we obtained the remission of anaemia in 17 out of 20 patients with mild side effects. In 15 patients a dose of 50 $UKg^{-1}$ three times a week was sufficient to maintain haemoglobin levels higher than 100 $gl^{-1}$. In one patient a dose of 75 $UKg^{-1}$ and in one patient a dose of 100 $UKg^{-1}$ needed, while three patients were considered non responders and required haemotransfusions.

Moreover it is of interest that our results seem to confirm data obtained by Miller (1992) on the lack of prediction of pretreatment erythropoietin levels to exogenous rHuEPO in patients with CDDP anaemia. In fact in our study two out of the three non responder patients presented the lowest levels of pretreatment serum erythropoietin. These data are consistent also with the reports by Ludwig (1990) and Oster (1990). The findings of the present study show the effectiveness and the safety of subcutaneous administration of rHuEPO even with a lower dose respect to that demonstrated effective by intravenous route. In fact while in Miller's study (1992) doses of 100 $UKg^{-1}$ and 200 $UKg^{-1}$ five times weekly offered the potentiation for optimal clinical response, in our study doses of 50–75 $UKg^{-1}$ three times a week were sufficient to obtain a clinical response.

For this reasons subcutaneous rHuEPO could be more convenient than intravenous administration, also considering the economic aspect. Considering that in our study 75 U $Kg^{-1}$ three times a week could be the optimal dose whereas in Miller's study the erythropoietin dose should be at least 100 U $Kg^{-1}$ five times a week, one week treatment requires the use of 225 $UKg^{-1}$ in our regimen and 500 $UKg^{-1}$ in Miller's regimen for each patient. Because in Italy the price of 1,000 U of rHuEPO is about $14 for hospital pharmacies intravenous regimen is surely more expensive.

On the basis of these data further trials seem to be recommended to define the optimal dose and route of administration in view of determining, by randomised studies, the real effect on transfusion requirements and chemotherapy administration.

### References

ALEXOUPOLOS, C.G., CHALEVELAKIS, G., KATSOULIS, C. & PALLIKARIS, G. (1986). Adverse effect of cis- diamminedichloroplatinum II (CDDP) on porphyrin metabolism in man. *Cancer Chemother. Pharmacol.*, **17**, 165–170.

CAZZOLA, M., PONCHIO, L., BEGUIN, Y., ROSTI, V., BERGAMASCHI, G., LIBERATO, N.L., FREGONI, V., NALLA, G., BAROSI, G. & ASCARI, E. (1992). Subcutaneous erythropoietin for treatment of refractory anemia in hematologic disorders. Results of a phase I/II clinical trial. *Blood*, **79**, 29–37.

ERSLEV, A.J. (1991). Erythropoietin *N. Engl. J. Med.*, **324**, 1339–1344.

ESCHBACH, J.W., KELLY, M.R., HALEY, N.R., ABELS, R.I. & ADAMSON, J.W. (1989). Treatment of the anemia of progressive renal failure with recombinant human erythropoietin. N. Engl. J. Med., **321**, 158–163.

GALE, R.P. (1985). Antineoplastic chemotherapy myelosuppression: mechanism and new approaches. *Exp. Hematol.*, **13**, (suppl. 16), 3–7.

LUDWIG, H., FRITZ, E., KOTZMAN, H., HOCKER, P., GISSLINGER, H. & BARNAS, U. (1990). Erythropoietin treatment of anemia associated with multiple myeloma. *N. Engl. J. Med.*, **32**, 1693–1699.

MATSUMOTO, T., ENDOH, K., KAMISANGO, K., AKAMATSU, K., KOIZUMI, K., HIGUCHI, M., IMAI, N., MITSUI, H. & KAWAGUCHI, T. (1990). Effect of recombinant human erythropoietin on anticancer drug-induced anaemia. *Br. J. Haematol.*, **75**, 463–468.

MCMAHON, F.G., VARGAS, R., RYAN, M., JAIN, A.K., ABELS, R.I., PERRY, B. & SMITH, I.L. (1990). Pharmacokinetics and effects of recombinant human erythropoietin after intravenous and subcutaneous injection in healthy volunteers. *Blood*, **76**, 1718–1722.

MILLER, C.B., JONES, R.J., PIANTADOSI, S., ABELOFF, M.D. & SPIVAK, J.L. (1990). Decreased erythropoietin response in patients with the anemia of cancer. *N. Engl. J. Med.*, **322**, 1689–1692.

MILLER, C.B., PLATANIAS, L.C., MILLS, S.R., ZAHURAK, M.L., RATAIN, M.J., ETTINGER, D.S. & JONES, R.J. (1992). Phase I-II trial of erythropoietin in the treatment of cisplatin-associated anemia. *J. Natl Cancer Inst.*, **84**, 98–103.

OSTER, W., HERRMANN, F., GAMM, H., ZEILE, H., LINDEMANN, A., MULLER, G., BRUNE, T., KRAEMER, H.P. & MERTELSMANN, R. (1990). Erythropoietin for the treatment of anemia of malignancy associated with neoplastic bone marrow infiltration. *J. Clin. Oncol.*, **8**, 956–962.

PLATANIAS, L.C., MILLER, C.B., MICK, R., HART, R.D., OZER, H., MCEVILLY, J.M., JONES, R.J. & RATAIN, M.J. (1991). Treatment of chemotherapy-induced anemia with recombinant human erythropoietin in cancer patients. *J. Clin. Oncol.*, **9**, 2021–2026.

ROSSOF, A.H., SLAYTON, R.E. & PERLIA, C.P. (1972). Preliminary clinical experience with cis-diamminedichloroplatinum(II) (NSC 119875, CACP). *Cancer*, **30**, 1451–1456.

ROTHMANN, S.A., PAUL, P., WEICK, J.K., MCINTYRE, W.R. & FANTELLI, F. (1985). Effect of cisdiamminedichloroplatinum on erytropoietin production and hematopoietic progenitor cells. *Int. J. Cell Cloning*, **3**, 415–423.

VON HOFF, D.D., SCHILSKY, R., REICHERT, C.M., REDDICK, R.L., ROZENCWEIG, M., YOUNG, R.C. & MUGGIA, F.M. (1979). Toxic effects of cisdichlorodiammineplatinum(II) in man. *Cancer Treat. Rep.*, **63**, 1527–1531.